# Comparison of various machine learning algorithms used for compressive strength prediction of steel fiber-reinforced concrete

**DOI:** 10.1038/s41598-023-30606-y

**Published:** 2023-03-04

**Authors:** Seyed Soroush Pakzad, Naeim Roshan, Mansour Ghalehnovi

**Affiliations:** Department of Civil Engineering, Faculty of Engineering, Ferdowsi University of Mashhad, Mashhad, Iran

**Keywords:** Civil engineering, Statistics, Composites

## Abstract

Adding hooked industrial steel fibers (ISF) to concrete boosts its tensile and flexural strength. However, the understanding of ISF’s influence on the compressive strength (CS) behavior of concrete is still questioned by the scientific society. The presented paper aims to use machine learning (ML) and deep learning (DL) algorithms to predict the CS of steel fiber reinforced concrete (SFRC) incorporating hooked ISF based on the data collected from the open literature. Accordingly, 176 sets of data are collected from different journals and conference papers. Based upon the initial sensitivity analysis, the most influential parameters like water-to-cement (W/C) ratio and content of fine aggregates (FA) tend to decrease the CS of SFRC. Meanwhile, the CS of SFRC could be enhanced by increasing the amount of superplasticizer (SP), fly ash, and cement (C). The least contributing factors include the maximum size of aggregates (D_max_) and the length-to-diameter ratio of hooked ISFs (L/D_ISF_). Several statistical parameters are also used as metrics to evaluate the performance of implemented models, such as coefficient of determination (R^2^), mean absolute error (MAE), and mean of squared error (MSE). Among different ML algorithms, convolutional neural network (CNN) with R^2^ = 0.928, RMSE = 5.043, and MAE = 3.833 shows higher accuracy. On the other hand, K-nearest neighbor (KNN) algorithm with R^2^ = 0.881, RMSE = 6.477, and MAE = 4.648 results in the weakest performance.

## Introduction

ML is a computational technique destined to simulate human intelligence and speed up the computing procedure by means of continuous learning and evolution. ML techniques have been effectively implemented in several industries, including medical and biomedical equipment, entertainment, finance, and engineering applications. ML can be used in civil engineering in various fields such as infrastructure development, structural health monitoring, and predicting the mechanical properties of materials. More specifically, numerous studies have been conducted to predict the properties of concrete^1–7^

One of the drawbacks of concrete as a fragile material is its low tensile strength and strain capacity. Hence, various types of fibers are added to increase the tensile load-bearing capability of concrete. To generate fiber-reinforced concrete (FRC), used fibers are typically short, discontinuous, and randomly dispersed throughout the concrete matrix^8^. Until now, fibers have been used mainly to improve the behavior of structural elements for serviceability purposes. However, the addition of ISF into the concrete and producing the SFRC may also provide additional strength capacity or act as the primary reinforcement in structural elements. Nowadays, For the production of prefabricated and in-situ concrete structures, SFRC is gaining acceptance such as (a) secondary reinforcement for temporary load scenarios, arresting shrinkage cracks, limiting micro-cracks occurring during transportation or installation of precast members (like tunnel lining segments), (b) partial substitution of the conventional reinforcement, i.e., hybrid reinforcement systems, and (c) total replacement of the typical reinforcement in compression-exposed elements, e.g., thin-shell structures, ground-supported slabs, foundations, and tunnel linings^9^. Unquestionably, one of the barriers preventing the use of fibers in structural applications has been the difficulty in calculating the FRC properties (especially CS behavior) that should be included in current design techniques^10^.

Accordingly, many experimental studies were conducted to investigate the CS of SFRC. Han et al.^11^ reported that the length of the ISF (L_ISF_) has an insignificant effect on the CS of SFRC. Setti et al.^12^ also introduced ISF with different volume fractions (V_ISF_) to the concrete and reported the improvement of CS of SFRC by increasing the content of ISF. Zhu et al.^13^ noticed a linearly increase of CS by increasing V_ISF_ from 0 to 2.0%. Despite the enhancement of CS of normal strength concrete incorporating ISF, no significant change of CS is obtained for high-performance concrete mixes by increasing V_ISF_^14,15^. This highlights the role of other mix’s components (like W/C ratio, aggregate size, and cement content) on CS behavior of SFRC. Therefore, owing to the difficulty of CS prediction through linear or nonlinear regression analysis, data-driven models are put into practice for accurate CS prediction of SFRC.

Recently, ML algorithms have been widely used to predict the CS of concrete. For instance, numerous studies^1–3,7,16,17^ have been conducted for predicting the mechanical properties of normal concrete (NC). Evidently, SFRC comprises a bigger number of components than NC including L_ISF_, L/D_ISF_, fiber type, diameter of ISF (D_ISF_) and the tensile strength of ISFs. In this regard, developing the data-driven models to predict the CS of SFRC is a comparatively novel approach. Kang et al.^18^ collected a datasets containing 7 features (V_ISF_ and L/D_ISF_ as the properties of fibers) and developed 11 various ML techniques and observed that the tree-based models had the best performance in predicting the CS of SFRC. Also, it was concluded that the W/C ratio and silica fume content had the most impact on the CS of SFRC. Mahesh et al.^19^ used ML algorithms on a 140-raw dataset considering 8 different features (L_ISF_, V_ISF_, and L/D_ISF_ as the fiber properties) and concluded that the artificial neural network (ANN) had the best performance in predicting the CS of SFRC with a regression coefficient of 0.97. Moreover, in a study conducted by Awolusi et al.^20^ only 3 features (L/D_ISF_ as the fiber properties) were considered, and ANN and the genetic algorithm models were implemented to predict the CS of SFRC. It was observed that overall, the ANN model outperformed the genetic algorithm in predicting the CS of SFRC.

According to the presented literature, the scientific community is still uncertain about the CS behavior of SFRC. In addition, the studies based on ML techniques that have been done to predict the CS of SFRC are limited since it is difficult to collect inclusive experimental data to develop models regarding all contributing features (such as the properties of fibers, aggregates, and admixtures). Hence, the presented study aims to compare various ML algorithms for CS prediction of SFRC based on all the influential parameters. For this purpose, 176 experimental data containing 11 features of SFRC are gathered from different journal papers. The primary sensitivity analysis is conducted to determine the most important features. Therefore, based on expert opinion and primary sensitivity analysis, two features (length and tensile strength of ISF) were omitted and only nine features were left for training the models. Then, nine well received ML algorithms are developed on the data and different metrics were used to evaluate the performance of these algorithms. Also, to prevent overfitting, the leave-one-out cross-validation method (LOOCV) is implemented, and 8 different metrics are used to assess the efficiency of developed models.

## Material and method

### Data collection

The SFRC mixes containing hooked ISF and their 28-day CS (tested by 150 mm cubic samples) were collected from the literature^11,13,21–33^. Some of the mixes were eliminated due to comprising recycled steel fibers or the other types of ISFs (such as smooth and wavy). Moreover, some others were omitted because of lacking the information of mixing components (such as FA, SP, etc.). Eventually, 63 mixes were omitted and 176 mixes were selected for training the models in predicting the CS of SFRC. All these mixes had some features such as D_MAX_, the amount of ISF (ISF), L/D_ISF_, C, W/C ratio, coarse aggregate (CA), FA, SP, and fly ash as input parameters (9 features). Also, the CS of SFRC was considered as the only output parameter.

### Compare the correlation between the variables

**Figure 1:**
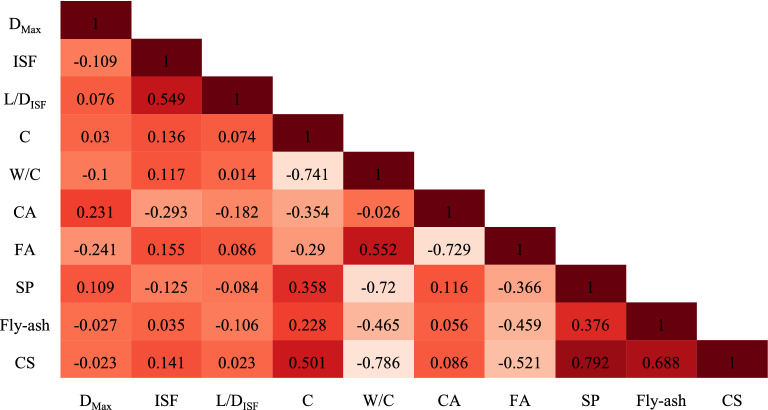
pair-wise correlation between variables.

**Figure 2:**
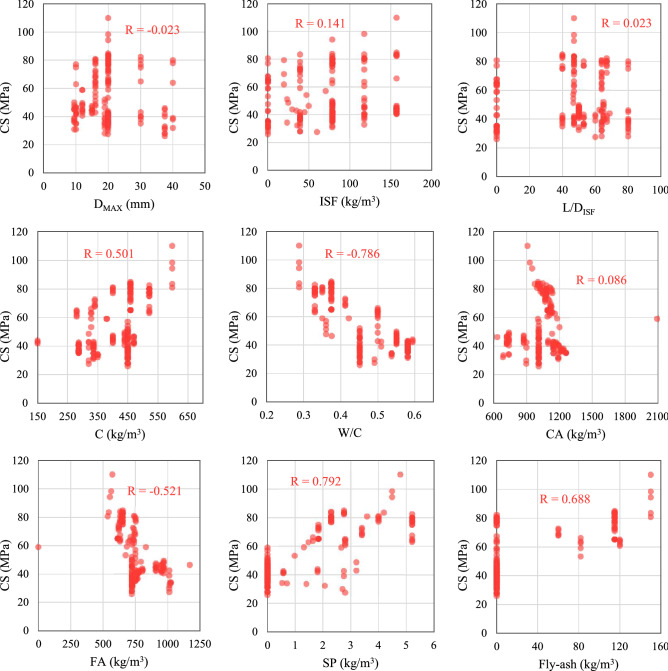
Correlation between numeric variables.

The correlation of all parameters with each other (pairwise correlation) can be seen in Fig. 1. Also, Fig. 2 illustrates the correlation between input parameters and the CS of SFRC.

The correlation coefficient ($R$) is a statistical measure that shows the strength of the linear relationship between two sets of data. Equation (1) is the covariance between two variables ($COV_{XY}$) divided by their standard deviations ($\sigma_{X}$, $\sigma_{Y}$). $R$ shows the direction and strength of a two-variable relationship. The linear relationship between two variables is stronger if $R$ is close to + 1.00 or − 1.00.

1 $$R_{XY} = \frac{{COV_{XY} }}{{\sigma_{X} \sigma_{Y} }}$$

As can be seen in Fig. 2, it is obvious that the CS increased with increasing the SP (R = 0.792) followed by fly ash (R = 0.688) and C (R = 0.501). Whereas, it decreased by increasing the W/C ratio (R = − 0.786) followed by FA (R = − 0.521). However, the CS of SFRC was insignificantly influenced by D_MAX_, CA, and properties of ISF (ISF, L/D_ISF_). The same results are also reported by Kang et al.^18^.

### Calibration

Statistical characteristics of input parameters, including the minimum, maximum, average, and standard deviation (SD) values of each parameter, can be observed in Table 1.

**Table 1:** Feature descriptions of SFRC mix proportions.

Parameters	Minimum	Maximum	Mean	SD
D_max_ (mm)	9.50	40.00	19.63	7.61
ISF (kg/m^3^)	0.00	157.00	70.44	49.84
L/D _ISF_	0.00	80.00	45.83	24.45
C (kg/m^3^)	150.00	598.00	405.38	87.09
W/C	0.29	0.59	0.47	0.09
CA (kg/m^3^)	632.00	2092.00	1036.23	164.62
FA (kg/m^3^)	0.00	1172.00	750.93	131.03
SP (kg/m^3^)	0.00	5.21	1.42	1.79
Fly-ash (kg/m^3^)	0.00	150.00	28.04	49.48

According to Table 1, input parameters do not have a similar scale. Therefore, the data needs to be normalized to avoid the dominance effect caused by magnitude differences among input parameters^34^. Normalization is a data preparation technique that converts the values in the dataset into a standard scale. It is essential to note that, normalization generally speeds up learning and leads to faster convergence. consequently, the max–min normalization method is adopted to reshape all datasets to a range from $0$ to $1$ using Eq. (2) as follows:

2 $$x_{norm} = \frac{{x - x_{\min } }}{{x_{\max } - x_{\min } }}$$

### Evaluation metrics

In some studies^34–37^, several metrics were used to sufficiently evaluate the performed models and compare their robustness. Accordingly, several statistical parameters such as R^2^, MSE, mean absolute percentage error (MAPE), root mean squared error (RMSE), average bias error (MBE), t-statistic test (T_stat_), and scatter index (SI) were used. R^2^ is a metric that demonstrates how well a model predicts the value of a dependent variable and how well the model fits the data. Various orders of marked and unmarked errors in predictions are demonstrated by MSE, RMSE, MAE, and MBE^6^. MAPE is a scale-independent measure that is used to evaluate the accuracy of algorithms. T_Stat_ and SI are the non-dimensional measures that capture uncertainty levels in the step of prediction. SI is a standard error measurement, whose smaller values indicate superior model performance. Evaluation metrics can be seen in Table 2, where $N$, $y_{i}$, $y_{i}^{\prime }$, and $\overline{y}$ represent the total amount of data, the true CS of the sample $i{\text{th}}$, the estimated CS of the sample $i{\text{th}}$, and the average value of the actual strength values, respectively.

**Table 2:** Metrics used to evaluate performance of given machine learning models.

Metric	Formula	Description
R^2^	$1 - \frac{{\sum\limits_{i = 1}^{N} {(y_{i} - y^{\prime}_{i} )^{2} } }}{{\sum\limits_{i = 1}^{N} {(y_{i} - \overline{y}_{i} )^{2} } }}$	Coefficient of determination
MSE	$\frac{1}{N}\sum\limits_{i = 1}^{N} {(y_{i} - y^{\prime}_{i} )^{2} }$	Mean squared error
RMSE	$\sqrt {\frac{1}{N}\sum\limits_{i = 1}^{N} {(y_{i} - y^{\prime}_{i} )^{2} } }$	Root mean squared error
MAE	$\frac{1}{N}\sum\limits_{i = 1}^{N} {\left| {y_{i} - y^{\prime}_{i} } \right|}$	Mean absolute error
MAPE	$\frac{1}{N}\sum\limits_{i = 1}^{N} {\left| {\frac{{y_{i} - y^{\prime}_{i} }}{{y_{i} }}} \right|}$	Mean absolute percentage error
MBE	$\frac{1}{N}\sum\limits_{i = 1}^{N} {(y_{i} - y^{\prime}_{i} )}$	Mean bias error
T_stat_	$\sqrt {\frac{{(N - 1)MBE^{2} }}{{RMSE^{2} - MBE^{2} }}}$	t-statistic test
SI	$\frac{RMSE}{{Mean(y_{i} )}}$	Scatter index

### Validation methods

**Figure 3:**
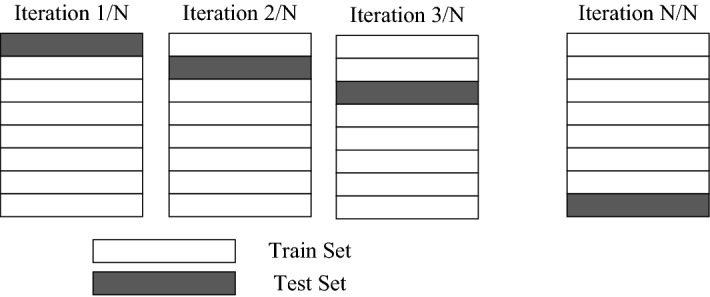
Leave-one-out cross-validation method.

To avoid overfitting, the dataset was split into train and test sets, with 80% of the data used for training the model and 20% for testing. Also, a specific type of cross-validation (CV) algorithm named LOOCV (Fig. 3) was used to validate the data and adjust the hyperparameters. In LOOCV, the number of folds is equal the number of instances in the dataset (n = 176).

### Implemented algorithms

As can be seen in Table 3, nine different algorithms were implemented in this research, including MLR, KNN, SVR, RF, GB, XGB, AdaBoost, ANN, and CNN.

**Table 3:** Applied models and their hyperparameters.

No.	Method	Details of hyperparameters
1	Multiple Linear Regression (MLR)	–
2	Random Forest (RF)	Number of estimators = 100; Maximum depth = 2
3	K Nearest Neighbors (KNN)	Algorithm = auto; leaf size = 40; number of jobs = − 1; number of neighbors = 7; *p* = 1; wights = uniform
4	Support Vector Regression (SVR)	Kernel = Radial Basis Function; C = 100; Gamma = 0.1; Epsilon = 0.01
5	Gradient Boosting (GB)	Number of estimators = 300; maximum depth = 3; learning rate = 0.01
6	Extreme Gradient Boosting (XGB)	Number of estimators = 500; maximum depth = 2; learning rate = 0.01; objective = squared error
7	Adaptive Boosting (AdaBoost)	Number of estimators = 200; learning rate = 1
8	Artificial Neural Network (ANN)	Architecture = Input: 9, 72, 72, 72, Output: 1 Dropout layers: 0.2, 0.2, 0.2, 0.2 Optimizer: Adam (Learning Rate: 0.01) Loss: Mean Squared Error Activation Function = (Hidden Layer: ReLU, Output Layer: Sigmoid) Batch Size: 20, Epochs: 50
9	Convolutional Neural Network (CNN)	Architecture: filter size layer one = 128; kernel size layer one = 2; max pooling size layer one = 2; Filter size layer two = 64; kernel size layer two = 2; max pooling size layer two = 2; Filter size layer three = 16; kernel size layer three = 2; average pooling size layer three = 2; Flatten layer neurons = 100; dropout layer (after flatten layer) = 0.2; learning rate = 0.01; batch size = 50; epochs = 20; Optimizer = Adam; loss function = mean squared error, activation function = ReLU
The hyper-parameters were adjusted using Grid Search and Random Search technique		

MLR is the most straightforward supervised ML algorithm for solving regression problems. Due to its simplicity, this model has been used to predict the CS of concrete in numerous studies^6,18,38,39^. MLR predicts the value of the dependent variable ($y$) based on the value of the independent variable ($x$) by establishing the linear relationship between inputs (independent parameters) and output (dependent parameter) based on Eq. (3):

3 $$\hat{y} = \alpha_{0} + \alpha_{1} x_{1} + \alpha_{2} x_{2} + \cdots + \alpha_{n} x_{n}$$

where $\hat{y}$, $x_{n}$, and $\alpha$ are the dependent parameter, independent parameter, and bias, respectively^18^.

The KNN method is a simple supervised ML technique that can be utilized in order to solve both classification and regression problems. This algorithm attempts to determine the value of a new point by exploring a collection of training sets located nearby^40^. This algorithm first calculates K neighbors’ euclidean distance. Then, among *K* neighbors, each category's data points are counted. Finally, the model is created by assigning the new data points to the category with the most neighbors.

**Figure 4:**
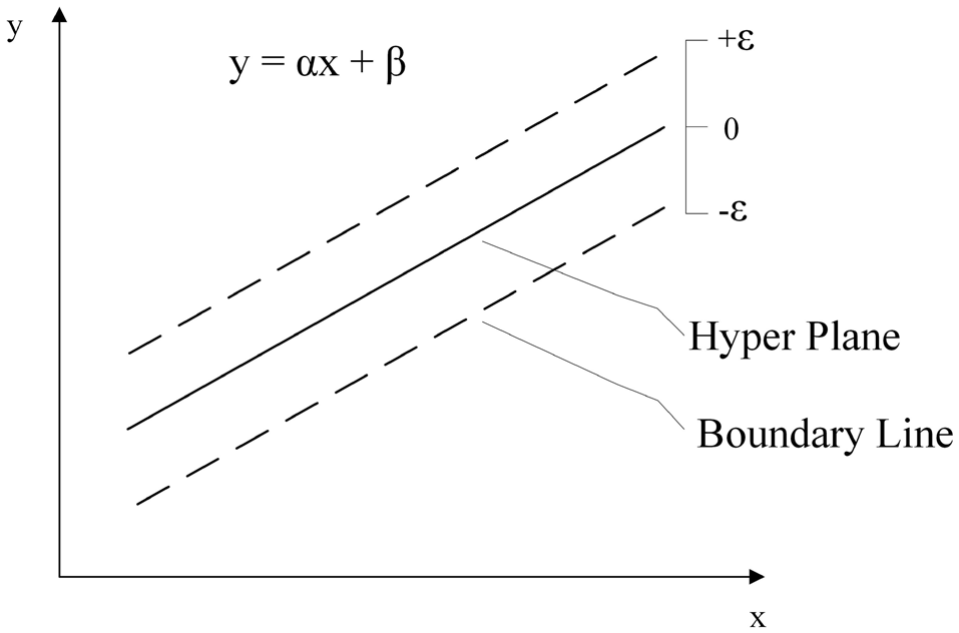
Support vector regression model.

SVR model (as can be seen in Fig. 4) has also been used to predict the CS of concrete^41,42^. SVR is considered as a supervised ML technique that predicts discrete values. In fact, SVR tries to determine the best fit line. The best-fitting line in SVR is a hyperplane with the greatest number of points. The primary rationale for using an SVR is that the problem may not be separable linearly. In these cases, an SVR with a non-linear kernel (e.g., a radial basis function) is used. In SVR, $\{ x_{i} ,y_{i} \} ,i = 1,2,...,k$ is the training set, where $x_{i}$ and $y_{i}$ are the input and output values, respectively. Moreover, the regression function is $y = \left\langle {\alpha ,x} \right\rangle + \beta$ and the aim of SVR is to flat the function as more as possible^18^.

All tree-based models can be applied to regression (predicting numerical values) or classification (predicting categorical values) problems. In the current research, tree-based models (GB, XGB, RF, and AdaBoost) were used to predict the CS of SFRC. Among these techniques, AdaBoost is the most straightforward boosting algorithm that is based on the idea that a very accurate prediction rule can be made by combining a lot of less accurate regulations^43^. Moreover, GB is an AdaBoost development model, a meta-estimator that consists of many sequential decision trees that uses a step-by-step method to build an additive model^6^. XGB makes GB more regular and controls overfitting by increasing the generalizability^6^. RF consists of many parallel decision trees and calculates the average of fitted models on different subsets of the dataset to enhance the prediction accuracy^6^.

**Figure 5:**
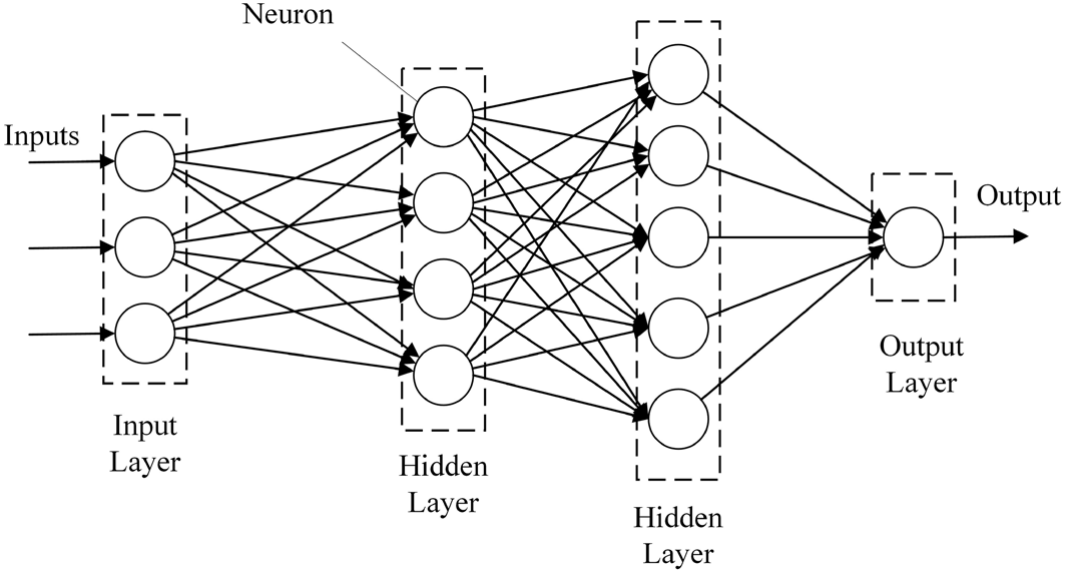
Artificial neural network model.

The use of an ANN algorithm (Fig. 5) as a powerful tool for estimating the CS of concrete is now well-known^6,38,44,45^. The brain’s functioning is utilized as a foundation for the development of ANN^6^. ANN can be used to model complicated patterns and predict problems. ANN model consists of neurons, weights, and activation functions^18^. the input values are weighted and summed using Eq. (4).

4 $$net_{j} = \sum\limits_{i = 1}^{n} {w_{ij} } x_{i} + b$$

where $x_{i} ,w_{ij} ,net_{j} ,$ and $b$ are the input values, the weight of each signal, the weighted sum of the $j{\text{th}}$ neuron, and bias, respectively^18^. In the current study, The ANN model was made up of one output layer and four hidden layers with 50, 150, 100, and 150 neurons each. There is a dropout layer after each hidden layer (The dropout layer sets input units to zero at random with a frequency rate at each training step, hence preventing overfitting). Adam was selected as the optimizer function with a learning rate of 0.01. It is essential to point out that the MSE approach was used as a loss function throughout the optimization process. Table 3 shows the results of using a grid and a random search to tune the other hyperparameters.

**Figure 6:**
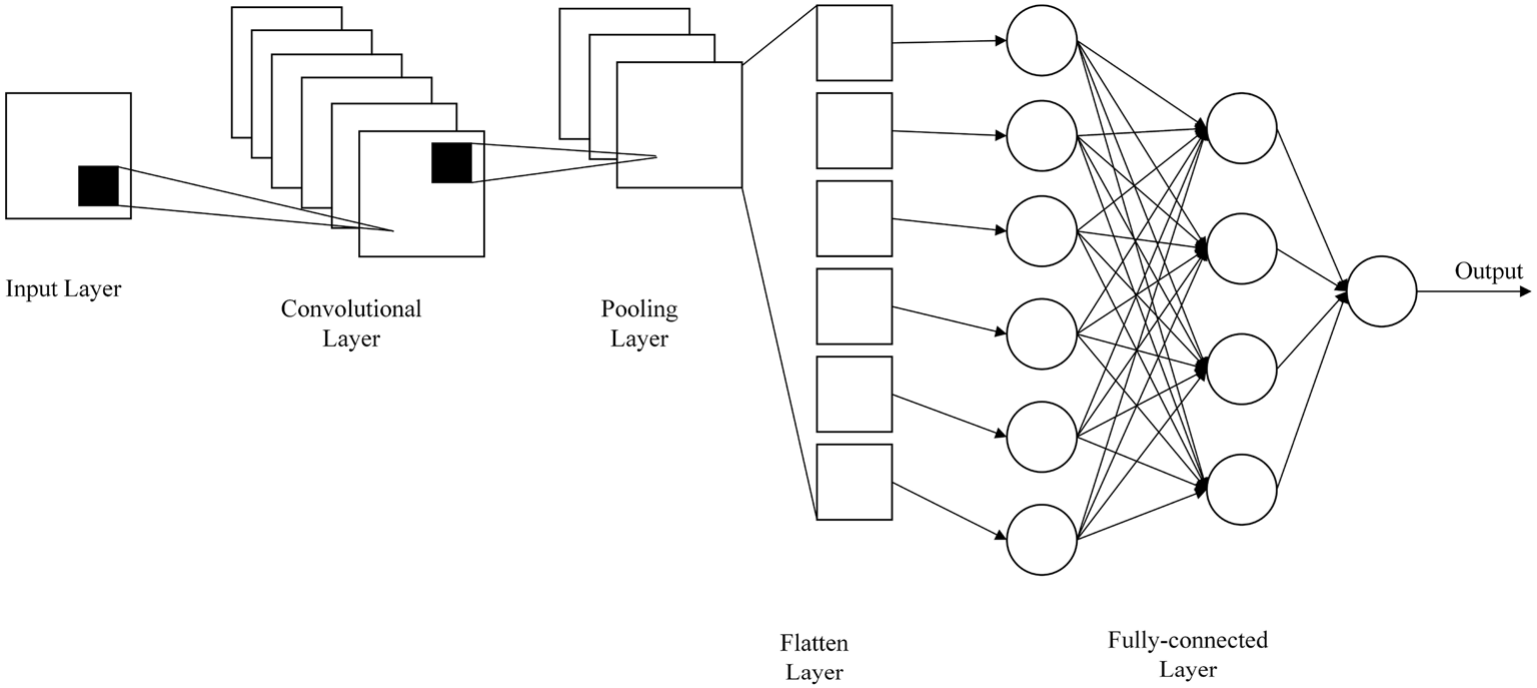
Convolutional neural network model.

In recent years, CNN algorithm (Fig. 6) has been increasingly used to predict the CS of concrete^34,46–49^. CNN model is a new architecture for DL which is comprised of several layers that process and transform an input to produce an output. In the current study, the architecture used was made up of a one-dimensional convolutional layer, a one-dimensional maximum pooling layer, a one-dimensional average pooling layer, and a fully-connected layer. Moreover, the ReLU was used as the activation function for each convolutional layer and the Adam function was employed as an optimizer. Table 3 displays the modified hyperparameters of each convolutional, flatten, hidden, and pooling layer, including kernel and filter size and learning rate.

### Tune hyperparameters

To adjust the validation set’s hyperparameters, random search and grid search algorithms were used. Table 3 provides the detailed information on the tuned hyperparameters of each model. The presented work uses Python programming language and the TensorFlow platform, as well as the Scikit-learn package.

## Result and discussion

**Figure 7:**
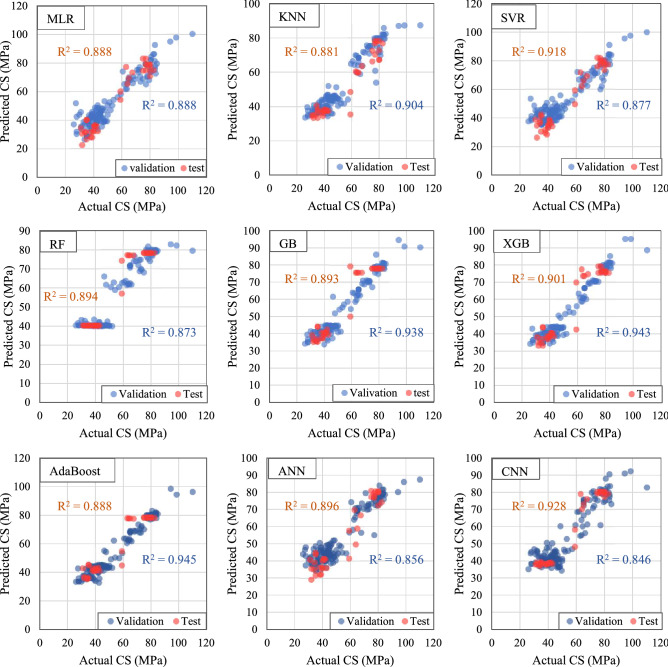
Performance of implimented algorithms in predicting CS of steel fiber-reinforced sconcrete (SFRC).

The CS of SFRC was predicted through various ML techniques as is described in section "Implemented algorithms". The predicted values were compared with the actual values to demonstrate the feasibility of ML algorithms (Fig. 7). As can be seen in Table 4, the performance of implemented algorithms was evaluated using various metrics.

**Table 4:** Performance parameters of applied models (Test set).

No.	Method	R^2^	MSE	RMSE	MAPE	MBE	MAE	T_stat_	SI
1	MLR	0.888	39.700	6.301	0.115	2.036	5.317	4.041	0.115
2	RF	0.894	37.489	6.123	0.094	− 3.372	4.430	7.806	0.112
3	KNN	0.881	41.950	6.477	0.086	3.337	4.648	7.112	0.119
4	SVR	0.918	29.126	5.397	0.102	1.210	4.559	2.723	0.099
5	GB	0.893	37.801	6.148	0.082	− 2.469	4.089	5.189	0.113
6	XGB	0.901	35.151	5.929	0.083	− 0.928	4.288	1.876	0.109
7	AdaBoost	0.888	39.560	6.290	0.092	− 2.606	4.431	5.388	0.115
8	ANN	0.896	36.680	6.056	0.093	1.057	4.383	2.097	0.111
9	CNN	0.928	25.432	5.043	0.078	− 1.757	3.833	4.398	0.092

As the simplest ML technique, MLR was implemented to predict the CS of SFRC and showed R^2^ of 0.888, RMSE of 6.301, and MAE of 5.317. Al-Abdaly et al.^50^ reported that MLR algorithm (with R^2^ = 0.64, RMSE = 8.68, MAE = 5.66) performed poorly in predicting the CS behavior of SFRC. Khademi et al.^51^ used MLR to predict the CS of NC and found that it cannot be considered an accurate model (with R^2^ = 0.518). Moreover, according to the results reported by Kang et al.^18^, it was shown that using MLR led to a significant difference between actual and predicted values for prediction of SFRC’s CS (RMSE = 12.4273, MAE = 11.3765). Hameed et al.^52^ developed an MLR model to predict the CS of high-performance concrete (HPC) and noted that MLR had a poor correlation between the actual and predicted CS of HPC (R = 0.789, RMSE = 8.288). Therefore, based on MLR performance in the prediction CS of SFRC and consistency with previous studies (in using the MLR to predict the CS of NC, HPC, and SFRC), it was suggested that, due to the complexity of the correlation between the CS and concrete mix properties, linear models (such as MLR) could not explain the complicated relationship among independent variables. So, more complex ML models such as KNN, SVR tree-based models, ANN, and CNN were proposed and implemented to study the CS of SFRC.

KNN (R^2^ = 0.881, RMSE = 6.477, MAE = 4.648) showed lower accuracy compared with MLR in predicting the CS of SFRC. Kang et al.^18^ observed that KNN predicted the CS of SFRC with a great difference between actual and predicted values. Asadi et al.^6^ also reported that KNN performed poorly in predicting the CS of concrete containing waste marble powder. Moreover, the CS of rubberized concrete was predicted using KNN algorithm by Hadzima-Nyarko et al.^53^, and it was reported that KNN might not be appropriate for estimating the CS of concrete containing waste rubber (RMSE = 8.725, MAE = 5.87). Therefore, according to the KNN results in predicting the CS of SFRC and compatibility with previous studies (in using the KNN in predicting the CS of various concrete types), it was observed that like MLR, KNN technique could not perform promisingly in predicting the CS of SFRC. This can refer to the fact that KNN considers all characteristics equally, even if they all contribute differently to the CS of concrete^6^.

Compared to the previous ML algorithms (MLR and KNN), SVR’s performance was better (R^2^ = 0.918, RMSE = 5.397, MAE = 4.559). Also, a significant difference between actual and predicted values was reported by Kang et al.^18^ in predicting the CS of SFRC (RMSE = 18.024). For the prediction of CS behavior of NC, Kabirvu et al.^5^ implemented SVR, and observed that SVR showed high accuracy (with R^2^ = 0.97). Whereas, Koya et al.^39^ and Li et al.^54^ reported that SVR showed a high difference between experimental and anticipated values in predicting the CS of NC. Based on the results obtained from the implementation of SVR in predicting the CS of SFRC and outcomes from previous studies in using the SVR to predict the CS of NC and SFRC, it was concluded that in some research, SVR demonstrated acceptable performance. In contrast, others reported that SVR showed weak performance in predicting the CS of concrete. This can be due to the difference in the number of input parameters.

Based upon the results in this study, tree-based models performed worse than SVR in predicting the CS of SFRC. However, it is worth noting that their performance in predicting the CS of SFRC was superior to that of KNN and MLR. Among these tree-based models, AdaBoost (with R^2^ = 0.888, RMSE = 6.29, MAE = 4.433) and XGB (with R^2^ = 0.901, RMSE = 5.929, MAE = 4.288) were the weakest and strongest models in predicting the CS of SFRC, respectively. As is reported by Kang et al.^18^, among implemented tree-based models, XGB performed superiorly in predicting the CS of SFRC. Al-Abdaly et al.^50^ also reported that RF (R^2^ = 0.88, RMSE = 5.66, MAE = 3.8) performed better than MLR (R^2^ = 0.64, RMSE = 8.68, MAE = 5.66) in predicting the CS of SFRC. Khan et al.^55^ also reported that RF (R^2^ = 0.96, RMSE = 3.1) showed more acceptable outcomes than XGB and GB with, an R^2^ of 0.9 and 0.95 in the prediction CS of SFRC, respectively. Moreover, Nguyen-Sy et al.^56^ and Rathakrishnan et al.^57^, after implementing the XGB, noted that the XGB was the best model for predicting the CS of NC. Therefore, based on tree-based technique outcomes in predicting the CS of SFRC and compatibility with previous studies in using tree-based models for predicting the CS of various concrete types (SFRC and NC), it was concluded that tree-based models (especially XGB) showed good performance.

It was observed that ANN (with R^2^ = 0.896, RMSE = 6.056, MAE = 4.383) performed better than MLR, KNN, and tree-based models (except XGB) in predicting the CS of SFRC, but its accuracy was lower than the SVR and XGB (in both validation and test sets) techniques. Mahesh et al.^19^ noted that after tuning the model (number of hidden layers = 20, activation function = Tansin Purelin), ANN showed superior performance in predicting the CS of SFRC (R^2^ = 0.95). Karahan et al.^58^ implemented ANN with the Levenberg–Marquardt variant as the backpropagation learning algorithm and reported that ANN predicted the CS of SFRC accurately (R^2^ = 0.96). Asadi et al.^6^ also used ANN in estimating the CS of NC containing waste marble powder (LOOCV was used to tune the hyperparameters) and reported that in the validation set, ANN was unable to reach an R^2^ as high as GB and XGB. However, ANN performed accurately in predicting the CS of NC incorporating waste marble powder (R^2^ = 0.97) in the test set. Finally, it is observed that ANN performs weaker than SVR and XGB in terms of R^2^ in the validation set due to the non-convexity of the multilayer perceptron's loss surface. Consequently, it is frequently required to locate a local maximum near the global minimum^59^. Hence, After each model training session, hold-out sample generalization may be poor, which reduces the R^2^ on the validation set ^6^. However, it is suggested that ANN can be utilized to predict the CS of SFRC.

Eventually, among all developed ML algorithms, CNN (with R^2^ = 0.928, RMSE = 5.043, MAE = 3.833) demonstrated superior performance in predicting the CS of SFRC. In comparison to the other discussed methods, CNN was able to accurately predict the CS of SFRC with a significantly reduced dispersion degree in the figures displaying the relationship between actual and expected CS of SFRC. Using CNN modelling, Chen et al.^34^ reported that CNN could show excellent performance in predicting the CS of the SFRS and NC. Deng et al.^47^ also observed that CNN was better at predicting the CS of recycled concrete (average relative error = 3.65) than other methods. Finally, results from the CNN technique were consistent with the previous studies, and CNN performed efficiently in predicting the CS of SFRC.

Table 4 indicates the performance of ML models by various evaluation metrics. It is observed that in comparison models with R^2^, MSE, RMSE, and SI, CNN shows the best result in predicting the CS of SFRC, followed by SVR, and XGB. In contrast, KNN shows the worst performance among developed ML models in predicting the CS of SFRC. Comparing implemented ML algorithms in terms of T_stat_, it is observed that XGB shows the best performance, followed by ANN and SVR in predicting the CS of SFRC. However, regarding the T_stat_, the outcomes show that CNN performance was approximately 58% lower than XGB. Comparing ML models with regard to MAE and MAPE, it is seen that CNN performs superior in predicting the CS of SFRC, followed by GB and XGB. On the other hand, MLR shows the highest MAE in predicting the CS of SFRC. In terms MBE, XGB achieved the minimum value of MBE, followed by ANN, SVR, and CNN.

**Figure 8:**
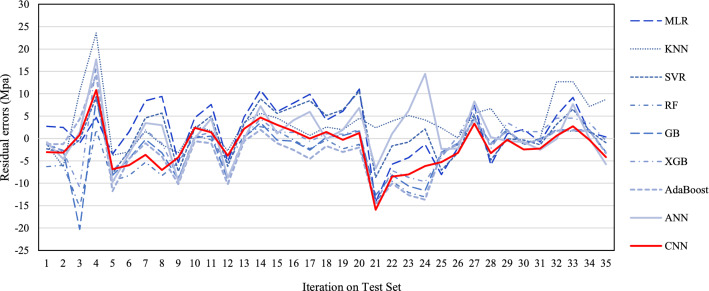
Fluctuations of errors (Actual CS–predicted CS) for different algorithms.

Figure 8 depicts the variability of residual errors (actual CS–predicted CS) for all applied models. If there is a lower fluctuation in the residual error and the residual errors fluctuate around zero, the model will perform better. Therefore, as can be perceived from Fig. 8, the SVR had the most outstanding performance and the least residual error fluctuation rate, followed by RF. In contrast, the XGB and KNN had the most considerable fluctuation rate. In addition, CNN achieved about 28% lower residual error fluctuation than SVR.

**Figure 9:**
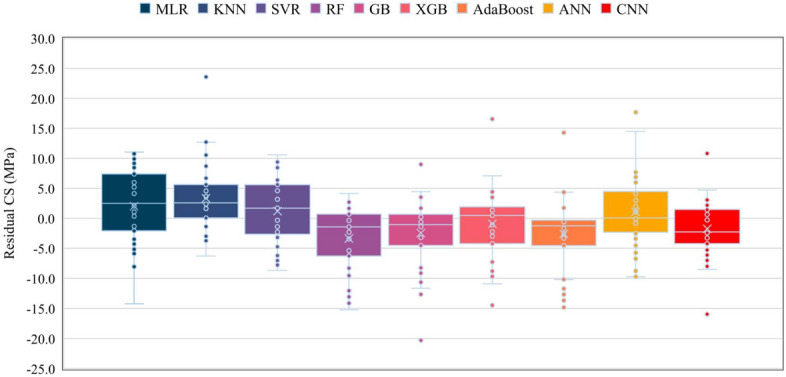
Distributions of errors in MPa (Actual CS–Predicted CS) for several methods.

As shown in Fig. 9, the minimum and maximum interquartile ranges (IQRs) belong to AdaBoost and MLR, respectively. In terms of comparing ML algorithms with regard to IQR index, CNN modelling showed an error dispersion about 31% lower than SVR technique. Moreover, CNN and XGB's prediction produced two more outliers than SVR, RF, and MLR's residual errors (zero outliers). Meanwhile, AdaBoost predicted the CS of SFRC with a broader range of errors.

**Figure 10:**
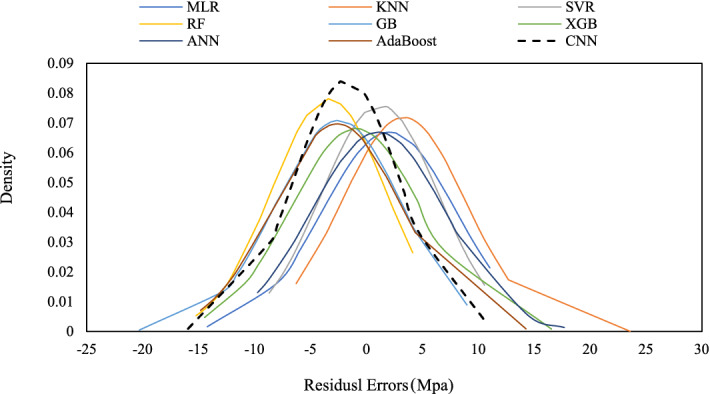
Normal distribution of errors (Actual CS–Predicted CS) for different methods.

Figure 10 also illustrates the normal distribution of the residual error of the suggested models for the prediction CS of SFRC. If a model's residual error distribution is closer to the normal distribution, there is a greater likelihood of prediction mistakes occurring around the mean value^6^. Based on this, CNN had the closest distribution to the normal distribution and produced the best results for predicting the CS of SFRC, followed by SVR and RF. Overall, it is possible to conclude that CNN produces more accurate predictions of the CS of SFRC with less uncertainty, followed by SVR and XGB.

### Sensitivity analysis

**Figure 11:**
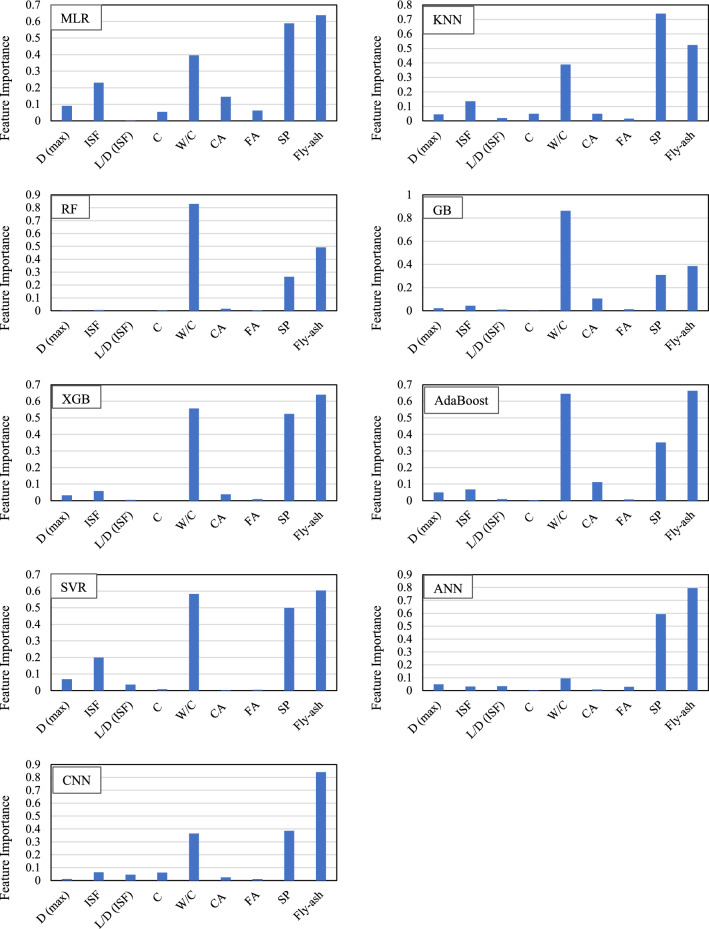
Feature importance of CS using various algorithms.

The sensitivity analysis investigates the importance's magnitude of input parameters regarding the output parameter. The feature importance of the ML algorithms was compared in Fig. 11. The sensitivity analysis demonstrated that, among different input variables, W/C ratio, fly ash, and SP had the most contributing effect on the CS behavior of SFRC, followed by the amount of ISF. Among these parameters, W/C ratio was commonly found to be the most significant parameter impacting the CS of SFRC (as the W/C ratio increases, the CS of SFRC will be increased). Knag et al.^18^ reported that silica fume, W/C ratio, and D_MAX_ are the most influential parameters that predict the CS of SFRC. Also, the characteristics of ISF (V_ISF_, L/D_ISF_) have a minor effect on the CS of SFRC. Li et al.^54^ noted that the CS of SFRC increased with increasing amounts of C and silica fume, and decreased with increasing amounts of water and SP. Therefore, based on the sensitivity analysis, the ML algorithms for predicting the CS of SFRC can be deemed reasonable.

### Parametric analysis

**Figure 12:**
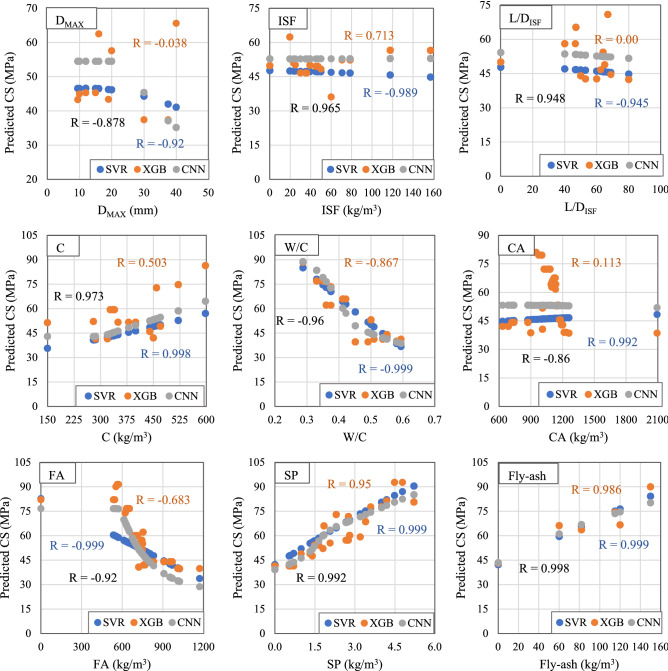
Parametric analysis between parameters and predicted CS in various algorithms.

A parametric analysis was carried out to determine how well the developed ML algorithms can predict the effect of various input parameters on the CS behavior of SFRC. To perform the parametric analysis to analyze the influence of one specific parameter (for example, W/C ratio) on the predicted CS of SFRC, the actual values of that parameter (W/C ratio) were considered, while the mean values for all the other input parameters values were introduced. The implemented procedure was repeated for other parameters as well, considering the three best-performed algorithms, which are SVR, XGB, and ANN. This method has also been used in other research works like the one Khan et al.^60^ did. The result of this analysis can be seen in Fig. 12.

As shown in Fig. 12, the W/C ratio is the parameter that intensively affects the predicted CS. In other words, the predicted CS decreases as the W/C ratio increases. Generally, the developed ML models can accurately predict the effect of the W/C ratio on the predicted CS. Moreover, among the three proposed ML models here, SVR demonstrates superior performance in estimating the influence of the W/C ratio on the predicted CS of SFRC with a correlation of R = − 0.999, followed by CNN with a correlation of R = − 0.96. The performance of the XGB algorithm is also reasonable by resulting in a value of R = − 0.867 for correlation.

In addition, Fig. 12 illustrates the impact of SP on the predicted CS of SFRC. As can be seen in Fig. 12, the SP has a medium impact on the predicted CS of SFRC. Moreover, among the proposed ML models, SVR performed better in predicting the influence of the SP on the predicted CS of SFRC with a correlation of R = 0.999, followed by CNN and XGB with a correlation of R = 0.992 and R = 0.95, respectively.

However, it is depicted that the weak correlation between the amount of ISF in the SFRC mix and the predicted CS. This indicates that the CS of SFRC cannot be predicted by only the amount of ISF in the mix. In other words, in CS prediction of SFRC, all the mixes’ components must be presented (such as the developed ML algorithms in the current study).

The impact of the fly-ash on the predicted CS of SFRC can be seen in Fig. 12. All three proposed ML algorithms demonstrate superior performance in predicting the correlation between the amount of fly-ash and the predicted CS of SFRC. It means that all ML models have been able to predict the effect of the fly-ash on the CS of SFRC. Moreover, it is essential to mention that only 26% of the presented mixes contained fly-ash, and the results obtained were according to these mixes. Therefore, these results may have deficiencies.

Based on the developed models to predict the CS of SFRC (Fig. 12), C, D_MAX_, L/D_ISF_, and CA have relatively little effect on the CS. Moreover, the results show that increasing the amount of FA causes a decrease in the CS of SFRC (Fig. 12). All these results are consistent with the outcomes from sensitivity analysis, which is presented in Fig. 11, and the correlation between input parameters and the CS of SFRC shown in Figs. 1 and 2.

## Conclusion

This study modeled and predicted the CS of SFRC using several ML algorithms such as MLR, tree-based models, SVR, KNN, ANN, and CNN. From the open literature, a dataset was collected that included 176 different concrete compressive test sets. This research leads to the following conclusions:

- Among the several ML techniques used in this research, CNN attained superior performance (R^2^ = 0.928, RMSE = 5.043, MAE = 3.833), followed by SVR (R^2^ = 0.918, RMSE = 5.397, MAE = 4.559). In contrast, KNN (R^2^ = 0.881, RMSE = 6.477, MAE = 4.648) showed the weakest performance in predicting the CS of SFRC.
- Tree-based models performed worse than SVR in predicting the CS of SFRC. However, their performance in predicting the CS of SFRC was superior to that of KNN and MLR.
- The capabilities of ML algorithms were demonstrated through a sensitivity analysis and parametric analysis. It was observed that among the concrete mixture properties, W/C ratio, fly-ash, and SP had the most significant effect on the CS of SFRC (W/C ratio was the most effective parameter). Also, C, D_MAX_, L/D_ISF_, and CA have relatively little effect on the CS of SFRC.
- According to the results obtained from parametric analysis, among the developed models, SVR can accurately predict the impact of W/C ratio, SP, and fly-ash on the CS of SFRC, followed by CNN.

## Data Availability

All data generated or analyzed during this study are included in this published article. The raw data is also available from the corresponding author on reasonable request.

## References

[1] Chou J-S, Pham A-D (2013). Enhanced artificial intelligence for ensemble approach to predicting high performance concrete compressive strength. Constr. Build. Mater..

[2] Chou J-S, Tsai C-F, Pham A-D, Lu Y-H (2014). Machine learning in concrete strength simulations: Multi-nation data analytics. Constr. Build. Mater..

[3] Duan J, Asteris PG, Nguyen H, Bui X-N, Moayedi H (2021). A novel artificial intelligence technique to predict compressive strength of recycled aggregate concrete using ICA-XGBoost model. Eng. Comput..

[4] Gupta S (2007). Support vector machines based modelling of concrete strength. World Acad. Sci. Eng. Technol..

[5] Kabiru, O. A., Owolabi, T. O., Ssennoga, T. & Olatunji, S. O. Performance comparison of SVM and ANN in predicting compressive strength of concrete (2014).

[6] Shamsabadi EA, Roshan N, Hadigheh SA, Nehdi ML, Khodabakhshian A, Ghalehnovi M (2022). Machine learning-based compressive strength modelling of concrete incorporating waste marble powder. Constr. Build. Mater..

[7] Young BA, Hall A, Pilon L, Gupta P, Sant G (2019). Can the compressive strength of concrete be estimated from knowledge of the mixture proportions?: New insights from statistical analysis and machine learning methods. Cem. Concr. Res..

[8] Behbahani, H., Nematollahi, B. & Farasatpour, M. Steel fiber reinforced concrete: A review (2011).

[9] Marcos-Meson V, Michel A, Solgaard A, Fischer G, Edvardsen C, Skovhus TL (2018). Corrosion resistance of steel fibre reinforced concrete-A literature review. Cem. Concr. Res..

[10] de Montaignac R, Massicotte B, Charron J-P, Nour A (2012). Design of SFRC structural elements: post-cracking tensile strength measurement. Mater. Struct..

[11] Han J, Zhao M, Chen J, Lan X (2019). Effects of steel fiber length and coarse aggregate maximum size on mechanical properties of steel fiber reinforced concrete. Constr. Build. Mater..

[12] Setti F, Ezziane K, Setti B (2020). Investigation of mechanical characteristics and specimen size effect of steel fibers reinforced concrete. J. Adhes. Sci. Technol..

[13] Zhu H, Li C, Gao D, Yang L, Cheng S (2019). Study on mechanical properties and strength relation between cube and cylinder specimens of steel fiber reinforced concrete. Adv. Mech. Eng..

[14] Lee S-C, Oh J-H, Cho J-Y (2015). Compressive behavior of fiber-reinforced concrete with end-hooked steel fibers. Materials.

[15] Ren G, Wu H, Fang Q, Liu J (2018). Effects of steel fiber content and type on static mechanical properties of UHPCC. Constr. Build. Mater..

[16] Deepa C, SathiyaKumari K, Sudha VP (2010). Prediction of the compressive strength of high performance concrete mix using tree based modeling. Int. J. Comput. Appl..

[17] Erdal HI (2013). Two-level and hybrid ensembles of decision trees for high performance concrete compressive strength prediction. Eng. Appl. Artif. Intell..

[18] Kang M-C, Yoo D-Y, Gupta R (2021). Machine learning-based prediction for compressive and flexural strengths of steel fiber-reinforced concrete. Constr. Build. Mater..

[19] Mahesh R, Sathyan D (2022). Modelling the hardened properties of steel fiber reinforced concrete using ANN. Mater. Today Proc..

[20] Awolusi T, Oke O, Akinkurolere O, Sojobi A, Aluko O (2019). Performance comparison of neural network training algorithms in the modeling properties of steel fiber reinforced concrete. Heliyon.

[21] Al-Baghdadi HM, Al-Merib FH, Ibrahim AA, Hassan RF, Hussein HH (2021). Effects of coarse aggregate maximum size on synthetic/steel fiber reinforced concrete performance with different fiber parameters. Buildings.

[22] Atiş CD, Karahan O (2009). Properties of steel fiber reinforced fly ash concrete. Constr. Build. Mater..

[23] Caggiano A, Folino P, Lima C, Martinelli E, Pepe M (2017). On the mechanical response of hybrid fiber reinforced concrete with recycled and industrial steel fibers. Constr. Build. Mater..

[24] Graeff, Â. G., Pilakoutas, K., Lynsdale, C. & Neocleous, K. Corrosion durability of recycled steel fibre reinforced concrete. *Intersect. Intersect.* **6**(4) (2009).

[25] Hu H, Papastergiou P, Angelakopoulos H, Guadagnini M, Pilakoutas K (2018). Mechanical properties of SFRC using blended manufactured and recycled tyre steel fibres. Constr. Build. Mater..

[26] Jamshidi Avanaki M, Abedi M, Hoseini A, Maerefat MS (2018). Effects of fiber volume fraction and aspect ratio on mechanical properties of hybrid steel fiber reinforced concrete. New Approaches Civ. Eng..

[27] Leone M, Centonze G, Colonna D, Micelli F, Aiello M (2018). Fiber-reinforced concrete with low content of recycled steel fiber: Shear behaviour. Constr. Build. Mater..

[28] Leone M, Centonze G, Colonna D, Micelli F, Aiello MA (2016). Experimental study on bond behavior in fiber-reinforced concrete with low content of recycled steel fiber. J. Mater. Civ. Eng..

[29] Martinelli E, Caggiano A, Xargay H (2015). An experimental study on the post-cracking behaviour of Hybrid Industrial/Recycled Steel Fibre-Reinforced Concrete. Constr. Build. Mater..

[30] Olivito R, Zuccarello F (2010). An experimental study on the tensile strength of steel fiber reinforced concrete. Compos. B Eng..

[31] Sanjeev J, Nitesh KS (2020). Study on the effect of steel and glass fibers on fresh and hardened properties of vibrated concrete and self-compacting concrete. Mater. Today Proc..

[32] Skarżyński Ł, Suchorzewski J (2018). Mechanical and fracture properties of concrete reinforced with recycled and industrial steel fibers using Digital Image Correlation technique and X-ray micro computed tomography. Constr. Build. Mater..

[33] Zhang Y, Gao L (2020). Influence of tire-recycled steel fibers on strength and flexural behavior of reinforced concrete. Adv. Mater. Sci. Eng..

[34] Chen H, Yang J, Chen X (2021). A convolution-based deep learning approach for estimating compressive strength of fiber reinforced concrete at elevated temperatures. Constr. Build. Mater..

[35] Dao DV, Ly H-B, Vu H-LT, Le T-T, Pham BT (2020). Investigation and optimization of the C-ANN structure in predicting the compressive strength of foamed concrete. Materials.

[36] Golafshani EM, Behnood A, Arashpour M (2020). Predicting the compressive strength of normal and High-Performance Concretes using ANN and ANFIS hybridized with Grey Wolf Optimizer. Constr. Build. Mater..

[37] Kandiri A, Golafshani EM, Behnood A (2020). Estimation of the compressive strength of concretes containing ground granulated blast furnace slag using hybridized multi-objective ANN and salp swarm algorithm. Constr. Build. Mater..

[38] Güçlüer K, Özbeyaz A, Göymen S, Günaydın O (2021). A comparative investigation using machine learning methods for concrete compressive strength estimation. Mater. Today Commun..

[39] Koya, B. P., Aneja, S., Gupta, R. & Valeo, C. Comparative analysis of different machine learning algorithms to predict mechanical properties of concrete. *Mech. Adv. Mater. Struct.* 1–18 (2021).

[40] de-Prado-Gil J, Palencia C, Silva-Monteiro N, Martínez-García R (2022). To predict the compressive strength of self compacting concrete with recycled aggregates utilizing ensemble machine learning models. Case Stud. Constr. Mater..

[41] Azimi-Pour M, Eskandari-Naddaf H, Pakzad A (2020). Linear and non-linear SVM prediction for fresh properties and compressive strength of high volume fly ash self-compacting concrete. Constr. Build. Mater..

[42] Tanyildizi H (2018). Prediction of the strength properties of carbon fiber-reinforced lightweight concrete exposed to the high temperature using artificial neural network and support vector machine. Adv. Civ. Eng..

[43] Schapire, R. E. Explaining adaboost. In *Empirical Inference: Festschrift in Honor of Vladimir N. Vapnik *37–52 (2013).

[44] Huang J, Liew J, Liew K (2021). Data-driven machine learning approach for exploring and assessing mechanical properties of carbon nanotube-reinforced cement composites. Compos. Struct..

[45] Song H (2021). Predicting the compressive strength of concrete with fly ash admixture using machine learning algorithms. Constr. Build. Mater..

[46] Abuodeh OR, Abdalla JA, Hawileh RA (2020). Assessment of compressive strength of Ultra-high Performance Concrete using deep machine learning techniques. Appl. Soft Comput..

[47] Deng F, He Y, Zhou S, Yu Y, Cheng H, Wu X (2018). Compressive strength prediction of recycled concrete based on deep learning. Constr. Build. Mater..

[48] Jang Y, Ahn Y, Kim HY (2019). Estimating compressive strength of concrete using deep convolutional neural networks with digital microscope images. J. Comput. Civ. Eng..

[49] Ly H-B, Nguyen T-A, Tran VQ (2021). Development of deep neural network model to predict the compressive strength of rubber concrete. Constr. Build. Mater..

[50] Al-Abdaly NM, Al-Taai SR, Imran H, Ibrahim M (2021). Development of prediction model of steel fiber-reinforced concrete compressive strength using random forest algorithm combined with hyperparameter tuning and k-fold cross-validation. East. Eur. J. Enterp. Technol..

[51] Khademi F, Akbari M, Jamal SM (2015). Prediction of compressive strength of concrete by data-driven models. I Manag. J Civ Eng.

[52] Hameed, M. M. & AlOmar, M. K. Prediction of compressive strength of high-performance concrete: Hybrid artificial intelligence technique. In*International Conference on Applied Computing to Support Industry: Innovation and Technology* 323–335 (Springer, 2019).

[53] Hadzima-Nyarko M, Nyarko EK, Lu H, Zhu S (2020). Machine learning approaches for estimation of compressive strength of concrete. Eur. Phys. J. Plus.

[54] Li Y (2022). Compressive strength of steel fiber-reinforced concrete employing supervised machine learning techniques. Materials.

[55] Khan K, Ahmad W, Amin MN, Ahmad A, Nazar S, Alabdullah AA (2022). Compressive strength estimation of steel-fiber-reinforced concrete and raw material interactions using advanced algorithms. Polymers.

[56] Nguyen-Sy T, Wakim J, To Q-D, Vu M-N, Nguyen T-D, Nguyen T-T (2020). Predicting the compressive strength of concrete from its compositions and age using the extreme gradient boosting method. Constr. Build. Mater..

[57] Rathakrishnan, V., Beddu, S. & Ahmed, A. N. Comparison studies between machine learning optimisation technique on predicting concrete compressive strength (2021).

[58] Karahan O, Tanyildizi H, Atis CD (2008). An artificial neural network approach for prediction of long-term strength properties of steel fiber reinforced concrete containing fly ash. J. Zhejiang Univ. Sci. A.

[59] Choromanska, A., Henaff, M., Mathieu, M., Arous, G. B. & LeCun, Y. The loss surfaces of multilayer networks. In *Artificial Intelligence and Statistics* 192–204. PMLR (2015)

[60] Khan MA, Memon SA, Farooq F, Javed MF, Aslam F, Alyousef R (2021). Compressive strength of fly-ash-based geopolymer concrete by gene expression programming and random forest. Adv. Civ. Eng..

